# (*E*)-*N*′-(2,4,5-Trimethoxy­benzyl­idene)isonicotinohydrazide dihydrate

**DOI:** 10.1107/S1600536810015254

**Published:** 2010-04-30

**Authors:** H. S. Naveenkumar, Amirin Sadikun, Pazilah Ibrahim, Chin Sing Yeap, Hoong-Kun Fun

**Affiliations:** a School of Pharmaceutical Sciences, Universiti Sains Malaysia, 11800 USM, Penang, Malaysia; b X-ray Crystallography Unit, School of Physics, Universiti Sains Malaysia, 11800 USM, Penang, Malaysia

## Abstract

The asymmetric unit of the title compound, C_16_H_17_N_3_O_4_·2H_2_O, contains one Schiff base mol­ecule and two water mol­ecules. The Schiff base mol­ecule exists in an *E* configuration with respect to the C=N double bond and is essentially planar, the dihedral angle between the benzene and pyridine rings being 5.48 (8)°. The three meth­oxy groups are also coplanar with the benzene ring [C—O—C—C torsion angles = 3.9 (2), 178.51 (15) and 0.8 (2) Å]. In the crystal structure, the water mol­ecules link the mol­ecules into a three-dimensional network *via* inter­molecular N—H⋯O, O—H⋯O, O—H⋯N and C—H⋯O hydrogen bonds.

## Related literature

For applications of isoniazid derivatives, see: Janin (2007 ▶); Maccari *et al.* (2005 ▶); Slayden & Barry (2000 ▶); Kahwa *et al.* (1986 ▶). For the preparation of the title compound, see: Lourenco *et al.* (2008 ▶). For related structures, see: Naveenkumar *et al.* (2009 ▶, 2010*a* ▶,*b* ▶); Shi (2005 ▶). For the stability of the temperature controller used for the data collection, see: Cosier & Glazer (1986 ▶).
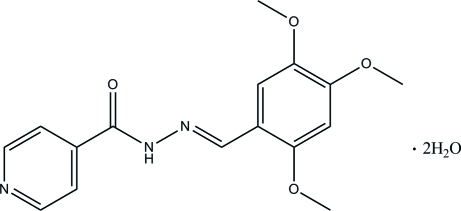


## Experimental

### 

#### Crystal data

C_16_H_17_N_3_O_4_·2H_2_O
*M* *_r_* = 351.36
Monoclinic, 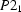

*a* = 6.8156 (4) Å
*b* = 14.5648 (10) Å
*c* = 8.5589 (5) Å
β = 103.421 (2)°
*V* = 826.42 (9) Å^3^
*Z* = 2
Mo *K*α radiation
μ = 0.11 mm^−1^
*T* = 100 K
0.50 × 0.28 × 0.19 mm

#### Data collection

Bruker APEXII DUO CCD area-detector diffractometer
Absorption correction: multi-scan (*SADABS*; Bruker, 2009 ▶) *T*_min_ = 0.947, *T*_max_ = 0.979
10676 measured reflections
2254 independent reflections
2171 reflections with *I* > 2σ(*I*)
*R*_int_ = 0.025

#### Refinement

*R*[*F* ^2^ > 2σ(*F* ^2^)] = 0.034
*wR*(*F* ^2^) = 0.126
*S* = 1.17
2254 reflections
233 parameters
1 restraint
H atoms treated by a mixture of independent and constrained refinement
Δρ_max_ = 0.57 e Å^−3^
Δρ_min_ = −0.56 e Å^−3^

### 

Data collection: *APEX2* (Bruker, 2009 ▶); cell refinement: *SAINT* (Bruker, 2009 ▶); data reduction: *SAINT*; program(s) used to solve structure: *SHELXTL* (Sheldrick, 2008 ▶); program(s) used to refine structure: *SHELXTL*; molecular graphics: *SHELXTL*; software used to prepare material for publication: *SHELXTL* and *PLATON* (Spek, 2009 ▶).

## Supplementary Material

Crystal structure: contains datablocks global, I. DOI: 10.1107/S1600536810015254/kj2145sup1.cif

Structure factors: contains datablocks I. DOI: 10.1107/S1600536810015254/kj2145Isup2.hkl

Additional supplementary materials:  crystallographic information; 3D view; checkCIF report

## Figures and Tables

**Table 1:** Hydrogen-bond geometry (Å, °)

*D*—H⋯*A*	*D*—H	H⋯*A*	*D*⋯*A*	*D*—H⋯*A*
N2—H1*N*2⋯O2*W*	0.88 (3)	2.05 (3)	2.916 (2)	171 (2)
O2*W*—H1*W*2⋯O1*W*	0.84	1.93	2.748 (2)	167
O2*W*—H2*W*2⋯N1^i^	0.83	2.10	2.887 (2)	158
O1*W*—H1*W*1⋯O3^ii^	0.85	2.18	2.8913 (19)	141
O1*W*—H1*W*1⋯O4^ii^	0.85	2.43	3.204 (2)	152
O1*W*—H2*W*1⋯O1^iii^	0.86	1.99	2.834 (2)	170
C4—H4*A*⋯O2*W*	0.93	2.34	3.253 (3)	169
C7—H7*A*⋯O2*W*	0.93	2.58	3.375 (2)	143
C14—H14*A*⋯O4^iii^	0.96	2.60	3.281 (2)	128

Symmetry codes: (i) 
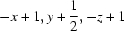
; (ii) 
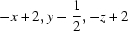
; (iii) 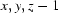.

**Fig. 1.**
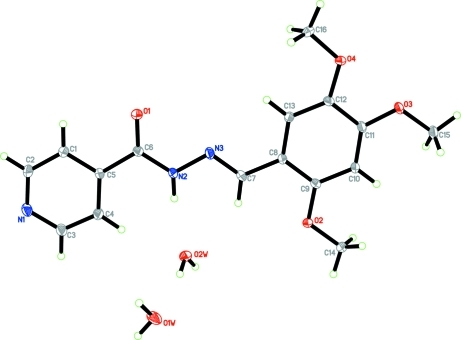
The molecular structure of the title compound with atom labels and 50% probability ellipsoids for non-H atoms.

**Fig. 2.**
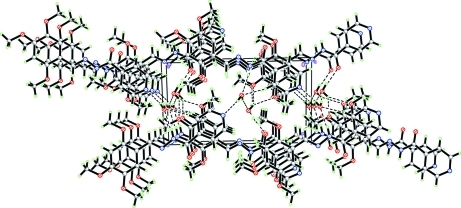
The crystal packing of the title compound, viewed down the a axis, showing the molecules linked into a 3-D network. Intermolecular hydrogen bonds are shown as dashed lines.

## supplementary crystallographic information

### Comment

In the search of new compounds, isoniazid derivatives have been found to possess potential tuberculostatic activity (Janin, 2007; Maccari *et al.*, 2005; Slayden & Barry, 2000). As a part of a current work of synthesis of such derivatives, in this paper we present the crystal structure of the title compound which was synthesized in our lab.

The asymmetric unit consists of one Schiff base molecule and two water molecules (Fig. 1). The geometry parameters are comparable to those related structures (Naveenkumar *et al.*, 2009, 2010a, b; Shi, 2005). The molecule exists in an *E* configuration with respect to the C7═N3 double bond. The molecule is essentially coplanar with dihedral angle between the benzene ring and the pyridine ring being 5.48 (8)°. The three methoxy groups are coplanar with the benzene ring [torsion angle, C14–O2–C9–C10 = 3.9 (2), C15–O3–C11–C12 = 178.51 (15), C16–O4–C12–C13 = 0.8 (2) Å]. In the crystal structure, the water molecules link the molecules into a three-dimensional network by the intermolecular N–H···O, O–H···O O–H···N and C–H···O hydrogen bonds (Fig. 2, Table 1).

### Experimental

The isoniazid derivative was prepared following the procedure by Lourenco *et al*., (2008). The title compound was prepared by reaction between 2, 4, 5-trimethoxybenzaldehyde (1.0 eq) and isoniazid (1.0 eq) in ethanol/water. After stirring for 1-3 hours at room temperature, the resulting mixture was concentrated under reduced pressure. The residue, purified by washing with cold ethanol and ethyl ether, afforded the pure derivative. The yellow single crystal suitable for X-ray analysis was obtained by recrystalization with methanol.

### Refinement

N-bound and O-bound hydrogen atoms were located from the difference Fourier map. The N-bound hydrogen atom was refined freely and the O-bound hydrogen atoms were constrained to ride on the parent atom with *U*_iso_(H) = 1.5 *U*_eq_(O). The rest of hydrogen atoms were positioned geometrically [C–H = 0.93 or 0.96 Å] and refined using a riding model, with *U*_iso_(H) = 1.2 or 1.5 *U*_eq_(C). Rotating-group models were applied for the methyl groups. As there is not enough anomalous dispersion to determine the absolute configuration, 4136 Friedel pairs were merged before final refinement.

### Crystal data

**Table:**

C_16_H_17_N_3_O_4_·2H_2_O	*F*(000) = 372
*M**_r_* = 351.36	*D*_x_ = 1.412 Mg m^−^^3^
Monoclinic, *P*2_1_	Mo *K*α radiation, λ = 0.71073 Å
Hall symbol: P 2yb	Cell parameters from 6099 reflections
*a* = 6.8156 (4) Å	θ = 3.1–37.4°
*b* = 14.5648 (10) Å	µ = 0.11 mm^−^^1^
*c* = 8.5589 (5) Å	*T* = 100 K
β = 103.421 (2)°	Block, yellow
*V* = 826.42 (9) Å^3^	0.50 × 0.28 × 0.19 mm
*Z* = 2	

### Data collection

**Table:**

Bruker APEXII DUO CCD area-detector diffractometer	2254 independent reflections
Radiation source: fine-focus sealed tube	2171 reflections with *I* > 2σ(*I*)
graphite	*R*_int_ = 0.025
φ and ω scans	θ_max_ = 29.0°, θ_min_ = 3.1°
Absorption correction: multi-scan (*SADABS*; Bruker, 2009)	*h* = −9→9
*T*_min_ = 0.947, *T*_max_ = 0.979	*k* = −19→19
10676 measured reflections	*l* = −11→11

### Refinement

**Table:**

Refinement on *F*^2^	Primary atom site location: structure-invariant direct methods
Least-squares matrix: full	Secondary atom site location: difference Fourier map
*R*[*F*^2^ > 2σ(*F*^2^)] = 0.034	Hydrogen site location: inferred from neighbouring sites
*wR*(*F*^2^) = 0.126	H atoms treated by a mixture of independent and constrained refinement
*S* = 1.17	*w* = 1/[σ^2^(*F*_o_^2^) + (0.098*P*)^2^] where *P* = (*F*_o_^2^ + 2*F*_c_^2^)/3
2254 reflections	(Δ/σ)_max_ < 0.001
233 parameters	Δρ_max_ = 0.57 e Å^−^^3^
1 restraint	Δρ_min_ = −0.56 e Å^−^^3^

### Special details

**Table:**

Experimental. The crystal was placed in the cold stream of an Oxford Cryosystems Cobra open-flow nitrogen cryostat (Cosier & Glazer, 1986) operating at 100.0 (1) K.
Geometry. All esds (except the esd in the dihedral angle between two l.s. planes) are estimated using the full covariance matrix. The cell esds are taken into account individually in the estimation of esds in distances, angles and torsion angles; correlations between esds in cell parameters are only used when they are defined by crystal symmetry. An approximate (isotropic) treatment of cell esds is used for estimating esds involving l.s. planes.
Refinement. Refinement of F^2^ against ALL reflections. The weighted R-factor wR and goodness of fit S are based on F^2^, conventional R-factors R are based on F, with F set to zero for negative F^2^. The threshold expression of F^2^ > 2sigma(F^2^) is used only for calculating R-factors(gt) etc. and is not relevant to the choice of reflections for refinement. R-factors based on F^2^ are statistically about twice as large as those based on F, and R- factors based on ALL data will be even larger.

### Fractional atomic coordinates and isotropic or equivalent isotropic displacement parameters (Å^2^)

**Table:**

	*x*	*y*	*z*	*U*_iso_*/*U*_eq_	
O1	0.7986 (2)	0.81175 (12)	1.11648 (15)	0.0180 (3)	
O2	0.6823 (2)	1.23398 (11)	0.79017 (15)	0.0156 (3)	
O3	0.8195 (2)	1.38828 (11)	1.30523 (16)	0.0189 (3)	
O4	0.89952 (19)	1.23697 (11)	1.45332 (14)	0.0162 (3)	
N1	0.6308 (2)	0.59240 (13)	0.6538 (2)	0.0172 (3)	
N2	0.7323 (2)	0.91163 (13)	0.90629 (17)	0.0130 (3)	
N3	0.7635 (2)	0.98623 (13)	1.00977 (19)	0.0138 (3)	
C1	0.7218 (3)	0.65940 (15)	0.9179 (2)	0.0171 (4)	
H1A	0.7568	0.6505	1.0285	0.020*	
C2	0.6821 (3)	0.58454 (16)	0.8141 (2)	0.0206 (4)	
H2A	0.6917	0.5260	0.8584	0.025*	
C3	0.6190 (3)	0.67728 (16)	0.5943 (2)	0.0170 (4)	
H3A	0.5837	0.6841	0.4833	0.020*	
C4	0.6562 (3)	0.75638 (15)	0.6879 (2)	0.0165 (4)	
H4A	0.6463	0.8141	0.6403	0.020*	
C5	0.7087 (2)	0.74703 (14)	0.8545 (2)	0.0118 (4)	
C6	0.7510 (2)	0.82624 (14)	0.9712 (2)	0.0126 (4)	
C7	0.7415 (2)	1.06540 (15)	0.9400 (2)	0.0127 (3)	
H7A	0.7100	1.0685	0.8285	0.015*	
C8	0.7652 (2)	1.14993 (14)	1.0336 (2)	0.0117 (3)	
C9	0.7321 (2)	1.23481 (15)	0.9547 (2)	0.0122 (4)	
C10	0.7488 (3)	1.31661 (14)	1.0429 (2)	0.0131 (4)	
H10A	0.7242	1.3727	0.9902	0.016*	
C11	0.8021 (2)	1.31332 (14)	1.2090 (2)	0.0130 (4)	
C12	0.8412 (2)	1.22870 (15)	1.2901 (2)	0.0128 (4)	
C13	0.8203 (2)	1.14806 (14)	1.2032 (2)	0.0116 (3)	
H13A	0.8428	1.0921	1.2566	0.014*	
C14	0.6607 (3)	1.32161 (15)	0.7124 (2)	0.0168 (4)	
H14A	0.6469	1.3133	0.5991	0.025*	
H14B	0.7777	1.3584	0.7552	0.025*	
H14C	0.5430	1.3519	0.7308	0.025*	
C15	0.7866 (3)	1.47619 (16)	1.2305 (2)	0.0184 (4)	
H15A	0.7906	1.5225	1.3110	0.028*	
H15B	0.6571	1.4771	1.1563	0.028*	
H15C	0.8900	1.4882	1.1738	0.028*	
C16	0.9457 (3)	1.15432 (15)	1.5425 (2)	0.0176 (4)	
H16A	1.0034	1.1687	1.6531	0.026*	
H16B	1.0403	1.1191	1.4999	0.026*	
H16C	0.8244	1.1193	1.5348	0.026*	
H1N2	0.692 (4)	0.923 (2)	0.803 (3)	0.017 (6)*	
O2W	0.6218 (2)	0.96947 (12)	0.57107 (16)	0.0189 (3)	
H1W2	0.7145	0.9636	0.5228	0.028*	
H2W2	0.5246	0.9958	0.5113	0.028*	
O1W	0.8961 (2)	0.92446 (13)	0.39361 (16)	0.0228 (3)	
H1W1	0.9693	0.8884	0.4600	0.034*	
H2W1	0.8554	0.8950	0.3055	0.034*	

### Atomic displacement parameters (Å^2^)

**Table:**

	*U*^11^	*U*^22^	*U*^33^	*U*^12^	*U*^13^	*U*^23^
O1	0.0273 (7)	0.0138 (8)	0.0110 (6)	0.0009 (5)	0.0009 (5)	0.0001 (5)
O2	0.0256 (6)	0.0115 (7)	0.0088 (6)	−0.0002 (5)	0.0017 (4)	0.0008 (5)
O3	0.0313 (7)	0.0101 (7)	0.0128 (6)	0.0004 (6)	0.0003 (5)	−0.0028 (5)
O4	0.0261 (6)	0.0122 (7)	0.0085 (5)	0.0014 (5)	0.0004 (4)	0.0006 (5)
N1	0.0194 (6)	0.0137 (9)	0.0176 (8)	−0.0008 (6)	0.0025 (5)	−0.0044 (6)
N2	0.0178 (6)	0.0097 (8)	0.0105 (6)	−0.0013 (6)	0.0016 (5)	−0.0023 (6)
N3	0.0161 (6)	0.0103 (8)	0.0143 (6)	−0.0011 (6)	0.0020 (5)	−0.0030 (6)
C1	0.0249 (8)	0.0123 (10)	0.0136 (8)	−0.0001 (7)	0.0035 (6)	0.0005 (7)
C2	0.0328 (9)	0.0099 (10)	0.0185 (9)	−0.0008 (8)	0.0045 (7)	−0.0008 (8)
C3	0.0202 (7)	0.0144 (10)	0.0148 (8)	0.0010 (7)	0.0010 (6)	−0.0023 (7)
C4	0.0214 (8)	0.0126 (10)	0.0139 (8)	0.0016 (7)	0.0008 (6)	0.0002 (7)
C5	0.0122 (6)	0.0100 (10)	0.0128 (7)	−0.0002 (6)	0.0025 (5)	−0.0016 (7)
C6	0.0132 (7)	0.0126 (10)	0.0115 (7)	−0.0002 (6)	0.0018 (5)	−0.0008 (6)
C7	0.0137 (6)	0.0121 (9)	0.0119 (7)	−0.0008 (6)	0.0024 (5)	−0.0031 (7)
C8	0.0138 (7)	0.0094 (9)	0.0118 (8)	−0.0003 (6)	0.0025 (5)	−0.0013 (6)
C9	0.0136 (7)	0.0125 (9)	0.0100 (7)	−0.0007 (7)	0.0017 (5)	−0.0017 (7)
C10	0.0169 (7)	0.0090 (9)	0.0129 (7)	−0.0001 (7)	0.0024 (6)	−0.0007 (7)
C11	0.0153 (7)	0.0098 (10)	0.0133 (8)	−0.0003 (7)	0.0020 (6)	−0.0026 (7)
C12	0.0145 (6)	0.0136 (10)	0.0095 (7)	0.0005 (7)	0.0010 (5)	−0.0003 (7)
C13	0.0126 (6)	0.0098 (9)	0.0121 (7)	−0.0006 (6)	0.0020 (5)	0.0006 (6)
C14	0.0252 (8)	0.0124 (10)	0.0123 (7)	0.0002 (7)	0.0034 (6)	0.0018 (7)
C15	0.0255 (8)	0.0096 (9)	0.0185 (8)	0.0006 (7)	0.0016 (6)	−0.0027 (7)
C16	0.0244 (8)	0.0154 (10)	0.0129 (7)	0.0018 (7)	0.0042 (6)	0.0042 (7)
O2W	0.0265 (6)	0.0172 (8)	0.0127 (6)	0.0040 (6)	0.0039 (5)	0.0029 (6)
O1W	0.0282 (7)	0.0261 (9)	0.0118 (6)	0.0080 (6)	0.0002 (5)	−0.0026 (6)

### Geometric parameters (Å, °)

**Table:**

O1—C6	1.228 (2)	C7—H7A	0.9300
O2—C9	1.3700 (19)	C8—C9	1.402 (3)
O2—C14	1.431 (2)	C8—C13	1.412 (2)
O3—C11	1.356 (2)	C9—C10	1.401 (3)
O3—C15	1.426 (3)	C10—C11	1.384 (2)
O4—C12	1.366 (2)	C10—H10A	0.9300
O4—C16	1.421 (2)	C11—C12	1.410 (3)
N1—C3	1.332 (3)	C12—C13	1.380 (3)
N1—C2	1.340 (3)	C13—H13A	0.9300
N2—C6	1.356 (3)	C14—H14A	0.9600
N2—N3	1.387 (2)	C14—H14B	0.9600
N2—H1N2	0.88 (3)	C14—H14C	0.9600
N3—C7	1.291 (3)	C15—H15A	0.9600
C1—C5	1.381 (3)	C15—H15B	0.9600
C1—C2	1.393 (3)	C15—H15C	0.9600
C1—H1A	0.9300	C16—H16A	0.9600
C2—H2A	0.9300	C16—H16B	0.9600
C3—C4	1.392 (3)	C16—H16C	0.9600
C3—H3A	0.9300	O2W—H1W2	0.8358
C4—C5	1.394 (2)	O2W—H2W2	0.8306
C4—H4A	0.9300	O1W—H1W1	0.8468
C5—C6	1.509 (3)	O1W—H2W1	0.8562
C7—C8	1.457 (3)		
			
C9—O2—C14	116.38 (15)	C10—C9—C8	120.44 (15)
C11—O3—C15	117.89 (14)	C11—C10—C9	119.55 (17)
C12—O4—C16	116.76 (16)	C11—C10—H10A	120.2
C3—N1—C2	116.66 (17)	C9—C10—H10A	120.2
C6—N2—N3	118.09 (14)	O3—C11—C10	124.14 (17)
C6—N2—H1N2	124 (2)	O3—C11—C12	115.12 (15)
N3—N2—H1N2	117 (2)	C10—C11—C12	120.74 (17)
C7—N3—N2	114.86 (15)	O4—C12—C13	126.55 (18)
C5—C1—C2	119.19 (17)	O4—C12—C11	113.82 (17)
C5—C1—H1A	120.4	C13—C12—C11	119.64 (15)
C2—C1—H1A	120.4	C12—C13—C8	120.47 (17)
N1—C2—C1	123.5 (2)	C12—C13—H13A	119.8
N1—C2—H2A	118.2	C8—C13—H13A	119.8
C1—C2—H2A	118.2	O2—C14—H14A	109.5
N1—C3—C4	124.15 (17)	O2—C14—H14B	109.5
N1—C3—H3A	117.9	H14A—C14—H14B	109.5
C4—C3—H3A	117.9	O2—C14—H14C	109.5
C3—C4—C5	118.48 (18)	H14A—C14—H14C	109.5
C3—C4—H4A	120.8	H14B—C14—H14C	109.5
C5—C4—H4A	120.8	O3—C15—H15A	109.5
C1—C5—C4	118.01 (17)	O3—C15—H15B	109.5
C1—C5—C6	117.48 (15)	H15A—C15—H15B	109.5
C4—C5—C6	124.52 (18)	O3—C15—H15C	109.5
O1—C6—N2	123.39 (17)	H15A—C15—H15C	109.5
O1—C6—C5	120.23 (18)	H15B—C15—H15C	109.5
N2—C6—C5	116.38 (15)	O4—C16—H16A	109.5
N3—C7—C8	120.95 (15)	O4—C16—H16B	109.5
N3—C7—H7A	119.5	H16A—C16—H16B	109.5
C8—C7—H7A	119.5	O4—C16—H16C	109.5
C9—C8—C13	119.13 (16)	H16A—C16—H16C	109.5
C9—C8—C7	119.69 (15)	H16B—C16—H16C	109.5
C13—C8—C7	121.18 (17)	H1W2—O2W—H2W2	109.2
O2—C9—C10	122.09 (17)	H1W1—O1W—H2W1	107.4
O2—C9—C8	117.47 (16)		
			
C6—N2—N3—C7	−179.38 (14)	C13—C8—C9—O2	178.90 (14)
C3—N1—C2—C1	0.2 (3)	C7—C8—C9—O2	−1.3 (2)
C5—C1—C2—N1	−0.1 (3)	C13—C8—C9—C10	−1.5 (2)
C2—N1—C3—C4	−0.1 (3)	C7—C8—C9—C10	178.21 (15)
N1—C3—C4—C5	−0.2 (3)	O2—C9—C10—C11	−179.21 (14)
C2—C1—C5—C4	−0.1 (3)	C8—C9—C10—C11	1.3 (2)
C2—C1—C5—C6	179.42 (16)	C15—O3—C11—C10	−1.9 (2)
C3—C4—C5—C1	0.3 (3)	C15—O3—C11—C12	178.51 (15)
C3—C4—C5—C6	−179.27 (16)	C9—C10—C11—O3	−179.05 (15)
N3—N2—C6—O1	−1.7 (2)	C9—C10—C11—C12	0.5 (2)
N3—N2—C6—C5	177.93 (14)	C16—O4—C12—C13	0.8 (2)
C1—C5—C6—O1	1.3 (2)	C16—O4—C12—C11	−178.67 (14)
C4—C5—C6—O1	−179.19 (16)	O3—C11—C12—O4	−2.9 (2)
C1—C5—C6—N2	−178.42 (15)	C10—C11—C12—O4	177.57 (14)
C4—C5—C6—N2	1.1 (2)	O3—C11—C12—C13	177.65 (15)
N2—N3—C7—C8	178.65 (14)	C10—C11—C12—C13	−1.9 (2)
N3—C7—C8—C9	−177.35 (15)	O4—C12—C13—C8	−177.80 (15)
N3—C7—C8—C13	2.4 (2)	C11—C12—C13—C8	1.6 (2)
C14—O2—C9—C10	3.9 (2)	C9—C8—C13—C12	0.1 (2)
C14—O2—C9—C8	−176.53 (14)	C7—C8—C13—C12	−179.66 (14)

### Hydrogen-bond geometry (Å, °)

**Table:**

*D*—H···*A*	*D*—H	H···*A*	*D*···*A*	*D*—H···*A*
N2—H1N2···O2W	0.88 (3)	2.05 (3)	2.916 (2)	171 (2)
O2W—H1W2···O1W	0.84	1.93	2.748 (2)	167
O2W—H2W2···N1^i^	0.83	2.10	2.887 (2)	158
O1W—H1W1···O3^ii^	0.85	2.18	2.8913 (19)	141
O1W—H1W1···O4^ii^	0.85	2.43	3.204 (2)	152
O1W—H2W1···O1^iii^	0.86	1.99	2.834 (2)	170
C4—H4A···O2W	0.93	2.34	3.253 (3)	169
C7—H7A···O2W	0.93	2.58	3.375 (2)	143
C14—H14A···O4^iii^	0.96	2.60	3.281 (2)	128

Symmetry codes: (i) −*x*+1, *y*+1/2, −*z*+1; (ii) −*x*+2, *y*−1/2, −*z*+2; (iii) *x*, *y*, *z*−1.
